# Role of Salt Concentration in Stabilizing Charged Ni-Rich Cathode Interfaces in Li-Ion Batteries

**DOI:** 10.1021/acs.chemmater.4c00004

**Published:** 2024-03-26

**Authors:** Conor M. E. Phelan, Erik Björklund, Jasper Singh, Michael Fraser, Pravin N. Didwal, Gregory J. Rees, Zachary Ruff, Pilar Ferrer, David C. Grinter, Clare P. Grey, Robert S. Weatherup

**Affiliations:** † Department of Materials, University of Oxford, Parks Road, Oxford OX1 3PH, U.K.; ‡ The Faraday Institution, Quad One, Harwell Science and Innovation Campus, Didcot OX11 0RA, U.K.; § Department of Chemistry, University of Cambridge, Lensfield Road, Cambridge CB2 1EW, U.K.; ∥ Diamond Light Source, Didcot, Oxfordshire OX11 0DE, U.K.; ⊥ Research Complex at Harwell, Didcot, Oxfordshire OX11 0DE, U.K.

## Abstract

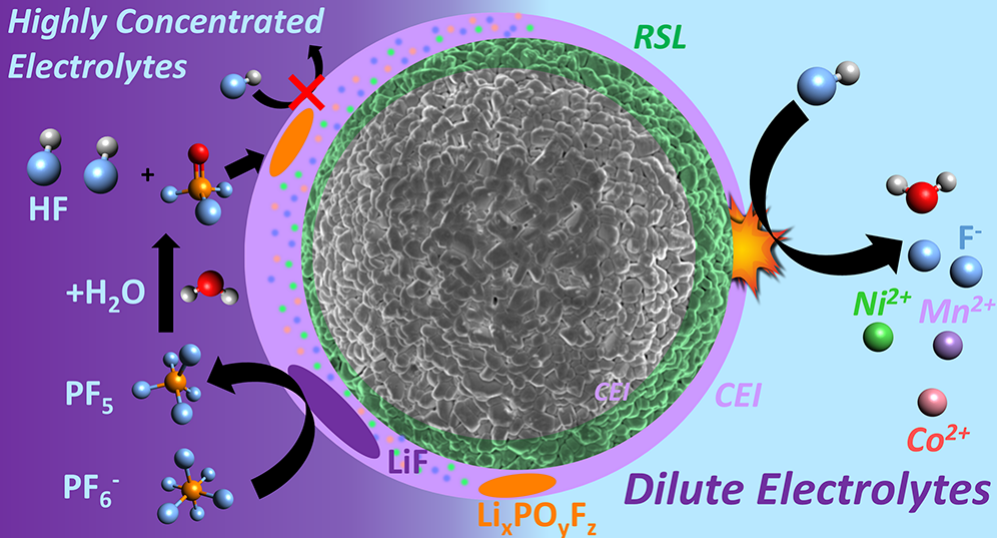

The cathode–electrolyte interphase (CEI) in Li-ion batteries plays a key role in suppressing undesired side reactions while facilitating Li-ion transport. Ni-rich layered cathode materials offer improved energy densities, but their high interfacial reactivities can negatively impact the cycle life and rate performance. Here we investigate the role of electrolyte salt concentration, specifically LiPF_6_ (0.5–5 *m*), in altering the interfacial reactivity of charged LiN_0.8_Mn_0.1_Co_0.1_O_2_ (NMC811) cathodes in standard carbonate-based electrolytes (EC/EMC vol %/vol % 3:7). Extended potential holds of NMC811/Li_4_Ti_5_O_12_ (LTO) cells reveal that the parasitic electrolyte oxidation currents observed are strongly dependent on the electrolyte salt concentration. X-ray photoelectron and absorption spectroscopy (XPS/XAS) reveal that a thicker Li_*x*_PO_*y*_F_*z*_-/LiF-rich CEI is formed in the higher concentration electrolytes. This suppresses reactions with solvent molecules resulting in a thinner, or less-dense, reduced surface layer (RSL) with lower charge transfer resistance and lower oxidation currents at high potentials. The thicker CEI also limits access of acidic species to the RSL suppressing transition-metal dissolution into the electrolyte, as confirmed by nuclear magnetic resonance (NMR) spectroscopy and inductively coupled plasma optical emission spectroscopy (ICP-OES). This provides insight into the main degradation processes occurring at Ni-rich cathode interfaces in contact with carbonate-based electrolytes and how electrolyte formulation can help to mitigate these.

## Introduction

Lithium-ion batteries (LIBs) play a vital role in the energy transition from fossil fuels toward the use of intermittent, renewable energy sources.^1^ Despite widespread use in electric vehicles and portable electronic devices, there is continuing demand to lower cost and further increase energy densities.^2^ Layered LiNi_1–*x*–*y*_Mn_*x*_Co_*y*_O_2_ (NMC) cathode materials are increasingly used in commercial LIBs, with the redox activity mainly associated with the Ni and Co centers. Increasing Ni content results in higher capacities with the additional benefit of lowering the content of Co, which is both expensive and has ethical concerns over its acquisition.^3^ However, this is accompanied by increased interfacial reactivities, particularly at high potentials, limiting the upper cutoff potentials that can be safely used.^4,5^ As NMC is delithiated at high potentials, its layered structure is destabilized, resulting in oxygen release and the formation of a reduced surface layer (RSL) at the particle surface.^6−10^ The RSL typically exhibits poor ionic conductivity, contributing to increased interfacial impedance,^7,10−12^ that limits high C-rate capability.

For more Ni-rich NMCs such as NMC811, the onset of significant RSL formation occurs at lower potentials vs Li/Li^+^,^8,9^ with the accompanying release of reactive ^1^O_2_ implicated in chemical oxidation of solvent components of the electrolyte.^1,8,9,13,14^ The associated formation of acidic species^8,15−21^ has been linked to transition metal (TM) dissolution from the cathode into the electrolyte and subsequent incorporation into the solid electrolyte interphase (SEI) on the anode,^4,5,10,21−23^ processes that are expected to be more prominent for Ni-rich NMCs and EC-containing electrolytes. Understanding the role of the electrolyte in these interfacial degradation mechanisms is critical to improving the cycle life of Ni-rich NMC cells to realize practical improvements in LIB energy density. The role of different organic carbonates in promoting certain degradation mechanisms at charged Ni-rich NMC cathodes has recently been explored.^4,5^ Here, we now investigate the role of LiPF_6_ concentration (0.5–5 *m*) using NMC811/LTO full cells in EC/EMC (vol %/vol % 3:7).

Cells were cycled such that the NMC811 reached a potential of 4.5 V vs Li/Li^+^ and were then held at this potential for 60 h, before discharge to 3.8 V vs Li/Li^+^. The cells were then characterized using ex situ XPS to determine CEI compositions, NMR spectroscopy to identify soluble electrolyte decomposition products, electrochemical impedance spectroscopy (EIS) to measure interfacial impedances, XAS to investigate the nature of the RSL and CEI on the NMC811, and ICP-OES to quantify the extent of TM dissolution. We thereby show that at higher electrolyte salt concentrations a thicker, more Li_*x*_PO_*y*_F_*z*_-/LiF-rich CEI is formed which protects the NMC811 against attack by solvent molecules and acidic species. This results in thinner/less-dense RSLs with lower charge transfer resistances, less TM dissolution into the electrolyte, and lower parasitic oxidation currents at high potentials. These findings provide insight into the role of the CEI in stabilizing the interfaces of Ni-rich NMCs in carbonate electrolytes, helping to inform strategies to mitigate NMC degradation at high potentials, such as through rational selection of electrolyte additives.

## Experimental Section

### Materials and Electrolyte Fabrication

NMC811 and LTO electrodes were purchased from NEI corporation with areal capacities of 1.15 and 1.5 mA h/cm^2^, respectively. The specific surface areas for the NMC811 and LTO electrodes were ≈0.35 and ≈7.0 m^2^/g, respectively. Both electrodes were composed of 90% active material, 5% carbon black, and 5% poly(vinylidene fluoride) (PVDF), with Al and Cu used as current collectors. Electrode discs were punched in an Ar-filled glovebox and dried under vacuum at 100 °C for 24 h in a Büchi oven.

Ethylene carbonate (EC, 99+%, Acros Organics) and ethyl methyl carbonate (EMC, 99.95+%, Solvionic) were mixed in a volume/volume ratio of 3:7 and dried for 24 h using dried molecular sieves (3 Å, beads, 8–12 mesh, Sigma-Aldrich). The water content of the EC/EMC mixture before mixing was measured using Karl Fischer titration and was found to be 3.3 ppm. Lithium hexafluorophosphate (LiPF_6_, 98+%, Fisher Scientific) was dried under dynamic vacuum at 1–2 mbar at 30 °C for 24 h. Electrolytes were mixed to differing molalities (0.5–5 *m*), with the EC/EMC mixture considered as the solvent. Glass fiber (GF) separators (borosilicate, grade GF/A, Whatman) were dried at 100 °C under dynamic vacuum at 1–2 mbar for 24 h prior to use.

### Electrochemical Cell Assembly and Protocols

2032-type coin cells (316 stainless steel, Cambridge Energy Solutions) were assembled in an Ar-filled glovebox from Ø 9.5 mm NMC811 cathodes, Ø 9.5 mm LTO anodes, and Ø 9.5 mm GF separators soaked in 60 μL of the electrolyte. A constant current of C/20 was applied to the coin cells until the potential across the full cell was 3.0 V, corresponding to the NMC811 reaching ≈4.5 V vs Li/Li^+^. The cell was held at this potential for 60 h and then discharged down to 2.3 V, corresponding to the NMC811 reaching 3.8 V vs Li/Li^+^. At least two cells were assembled for each measurement to ensure reproducibility. These coin cells were then disassembled and used in subsequent XPS, XAS, NMR, and ICP-OES measurements.

Three-electrode Swagelok-type PFA (Perfluoroalkoxy alkane) cells with 316 stainless steel (Taybroh Alloys) plungers were assembled with a Ø 9.5 mm NMC811 cathode and a Ø 9.5 mm LTO anode with a Ø 9.5 mm GF separator soaked in 60 μL of electrolyte between them. Li metal was used as the reference electrode and was separated from the anode and cathode with another Ø 9.5 mm GF separator soaked in 60 μL of electrolyte. The specific capacity of NMC811 was assumed to be 190 mA h g^–1^ when cycled between 3 and 4.3 V vs Li/Li^+^ as indicated by the manufacturer. Upon assembly, a constant current of C/20 was applied to the Swagelok-type cells until the potential of the NMC811 was 4.5 V vs Li/Li^+^. Each cell was held at this potential for 60 h and then discharged down to 3.8 V vs Li/Li^+^. EIS was then performed with the NMC811 at increasing potentials between 3.8 and 4.6 V vs Li/Li^+^ in steps of 0.1 V. EIS was performed in a frequency range of 500 kHz to 10 mHz with a peak-to-peak sinusoidal voltage perturbation of 5 mV. During EIS measurement, the potential of the NMC811 was not held at a constant value and a very small potential decay over the course of the measurement was observed.

We note that the potentials of Li incorporation are not fixed on the absolute potential scale and are expected to vary with salt concentration.^24^ However, from the Nernst equation, these potentials should all shift by the same amount for a given Li-ion activity, that is, salt concentration, leading to no relative change. Indeed, a constant potential difference between the Li and LTO electrodes was observed for all salt concentrations in our three-electrode cell measurements.

To investigate the effect of a preformed CEI, three-electrode Swagelok-type PFA cells were assembled in the same way and were subjected to a 60 h potential hold in the 3 *m* electrolyte. The NMC811 electrodes were recovered and reassembled into fresh three-electrode Swagelok-type PFA cells vs LTO in 0.5 *m* electrolyte and then subjected to a second 60 h potential hold.

### Materials Characterization

The NMC811/LTO coin cells were disassembled in an Ar-filled glovebox. The GF separator was extracted and soaked in 1 mL of deuterated DMSO (DMSO-*d*_6_) for 10 min. The solution was transferred into an airtight J. Young NMR tube and measured using an Oxford Instruments XPulse NMR spectrometer equipped with a 1.41 T magnet (ν_0_^1^H = 60 MHz). ^1^H and ^19^F NMR spectra were acquired for each of the different electrolyte concentrations, following the potential holds. All spectra were recorded, locked, and referenced to DMSO-*d*_6_.

The NMC811 and LTO electrodes were extracted and rinsed with dimethyl carbonate (DMC 99.99% anhydrous, Sigma-Aldrich). The electrodes were then vacuum-dried for 12 h at ambient temperature prior to inert transfer to the XPS equipment. XPS was performed using a PHI Versaprobe III XPS system generating focused, monochromatic Al Kα X-rays at 1486.6 eV, under ultrahigh vacuum (UHV) conditions (<10^–8^ mbar). A Shirley-type background was subtracted from all spectra, except for the Li 1s/TM 3p region. The probing depth, *d*, corresponding to the intensity contribution of 95% of the emitted photoelectrons was calculated as
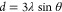
where λ is the inelastic mean free path and θ is the angle between the sample surface and the analyzer.^25^ An inelastic mean free path of 3.2 nm was calculated for photoelectrons emitted from the O 1s core level, assuming that they travel through a model interphase consisting of polyethylene.^26^ This assumes that the surface is flat and the chemical composition is homogeneous, which, although not the case for the composite electrodes used in this study, nevertheless serves as a reasonable approximation. The binding energies for each NMC811 sample were calibrated such that the C 1s peak of adventitious carbon (C–C) occurs at 285.0 eV.

Cycled NMC811 electrodes were inertly transferred to endstation 2 of beamline B07B (Diamond Light Source) for XAS measurements in both fluorescence yield (FY) and total electron yield (TEY) mode.^27^ FY involves the detection of photons with attenuation lengths of several hundred nm and thus provides more bulk-sensitive information on the electrodes’ oxidation states, while TEY involves detection of electrons emitted from the sample surface and thus a maximum probing depth of ≈10 nm.^28,29^ Samples were loaded onto a sample stage in an Ar glovebox prior to inert transfer to the endstation. Reference NMC electrode spectra from the literature were used to energy calibrate the TM L-edges.^10,30^ The O and F K-edges were energy-calibrated to NiO and LiF, respectively.^31^ Spectra are normalized to the incident photon flux and measured using a biased collector plate opposite the surface of the final refocusing mirror. For some low-intensity edges, sudden steps in the spectra related to periodic storage ring top-ups were removed during the data analysis.

Elemental analysis was performed by using ICP-OES (Thermo Scientific). The cycled LTO electrodes and GF separators were placed in borosilicate glass vials. 250 μL of concentrated nitric acid (66–68%) and 750 μL of ultrapure water (Millipore) were then added to the vials. After 3 days, the digested samples were diluted to 10 mL with additional ultrapure water and (in the case of the GF separator) centrifuged before measuring using ICP-OES. Calibration lines were generated for each element of interest from a concentration series made from a multielement standard solution at each wavelength of interest. Emission wavelengths were chosen such that there was no interference from other elements in the sample, the standard, or the matrix solution (2% nitric acid). The separator was carefully peeled away from the NMC811 electrode to minimize the amount of TMs inadvertently transferred between them on removal.

## Results

### Electrochemistry

**Figure 1:**
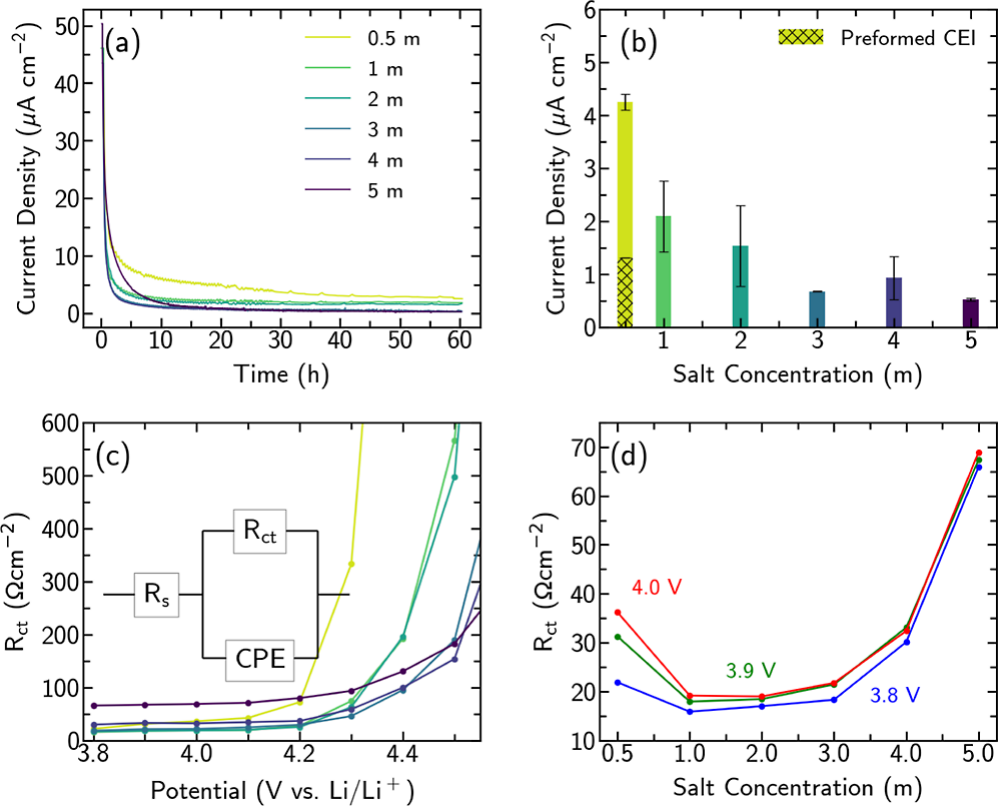
Comparison of electrochemical measurements in three-electrode Swagelok-type cells with different electrolyte salt concentrations from 0.5 to 5 *m*. (a) Current density profiles during 60 h potential holds at 4.5 V vs Li/Li^+^. (b) Average oxidation current density during the final 20 h of potential holds in different electrolyte salt concentrations, based on two or more cells with error bars indicating the standard deviation; and for a cell with a CEI preformed by holding in 3 *m* electrolyte, which is then held in 0.5 *m* electrolyte. (c) Variation in *R*_ct_ with potential following 60 h potential holds. (d) Variation of *R*_ct_ with salt concentration at different measurement potentials [same data as plotted in (c)].

Figure 1a shows the evolution of oxidation current density during 60 h potential holds (4.5 V vs Li/Li^+^) for three-electrode Swagelok-type cells containing different salt concentrations (0.5–5 *m*). The hold potential was selected to be above the onset of significant lattice oxygen release and accompanying RSL formation that is observed with commercial LP57 electrolytes.^1,8−10,13,14^ The initial current decay is largely attributable to relaxation of the electrolyte concentration polarization and the distribution of Li in the NMC811 particles as equilibrium is approached.^4^ The current density is expected to become increasingly dominated by electrolyte oxidation reactions, and thus, the average current density in the last 20 h provides a measure of the electrolyte stability at the charged Ni-rich interface. Figure 1b shows that these average oxidation current densities decrease for the electrolytes with higher salt concentrations, indicating improved stability at the NMC811 interface.

To probe how interfacial transport is altered by the electrolyte composition, potential-dependent EIS following the 60 h potential holds was performed in the same cells controlling the potential of the NMC811 vs Li/Li^+^ using the Li metal reference electrode with LTO as the counter electrode. Charge transfer resistances, *R*_ct_, at the NMC811 surface were extracted from Nyquist plots by fitting the intermediate frequency portion to the simple equivalent circuit in Figure 1c as demonstrated in Figure S1 (see the Supporting Information).^4,12,32^Figure 1c,d displays the dependence of *R*_ct_ on the electrode potential and electrolyte salt concentration, respectively. In considering these variations, the following expression obtained by assuming first-order insertion kinetics proves insightful (see Supporting Information for derivation).^32−35^
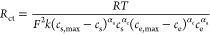
1*R* is the universal gas constant, *T* is temperature, *F* is Faraday’s constant, and *k* is an Arrhenius-type rate constant. *c*_s_ is the solid concentration of Li in the electrode, *c*_s,max_ is the solid concentration of Li in the fully lithiated electrode, *c*_e_ is the local electrolyte Li^+^ concentration, and *c*_e,max_ is the Li^+^ concentration at the solubility limit of the electrolyte, with α_a_ and α_c_ the anodic and cathodic transfer coefficients, respectively. This indicates that *R*_ct_ possesses contributions from the state of charge (SoC) of the electrode  as well as the local electrolyte Li^+^ concentration, .

From Figure 1c, a clear SoC dependence is apparent, with *R*_ct_ dramatically increasing at high potentials as the number of occupied Li sites in the delithiated electrode (*c*_s_) becomes negligible, typically referred to as a blocking condition. Significantly, the potential at which *R*_ct_ dramatically increases varies with the different electrolyte concentrations. For lower concentration electrolytes (0.5, 1, 2 *m*), this occurs between 4.2 and 4.4 V vs Li/Li^+^, while the higher concentration electrolytes (3, 4, 5 *m*) maintain lower *R*_ct_ at high potentials, with the rapid *R*_ct_ increase delayed to ≥4.4 V. These trends are attributable to differences in the extent of RSL formation at the NMC811 surface. Oxygen loss and TM migration during RSL formation lead to increased occupation of Li sites in the layered NMC by TM^2+^ ions,^36−39^ thereby lowering the number of Li-containing sites, *c*_s,max_, such that the blocking condition is reached at lower potentials. Although *k* is typically assumed to be constant with potential, it may be affected by the nature of RSL and thereby also influence *R*_ct_. Higher *R*_ct_ is typically associated with more extensive RSL formation,^7,10−12,40^ and thus the trends observed in Figure 1c indicate that thinner/less-densified RSLs are formed in the higher concentration electrolytes.

Figure 1d considers the variation in *R*_ct_ with salt concentration at intermediate potentials where the SoC dependence is not expected to dominate. For each fixed potential, a U-shaped curve is apparent that broadly follows the behavior expected based on eq 1, with *R*_ct_ increasing for the lowest and highest salt concentrations. Note that some SoC-dependent behavior is still apparent for the 0.5 *m* electrolyte. The increase in *R*_ct_ seen for the higher concentration electrolytes can be attributed to the electrolyte approaching its solubility limit such that (*c*_e,max_ – *c*_e_) becomes small, although Morasch et al. have observed changes in the rate constant *k* above ≈2.5 *m* which may also contribute.^32^ Similar trends in *R*_ct_ are observed for measurements of NMC811 that has undergone no potential hold so as to minimize RSL formation (see Figure S2); however, there are still notable differences to Figure 1d, likely related to the presence of the RSL and increased surface area associated with NMC particle cracking during the potential holds.

In addition to the RSL, the CEI formed by electrolyte decomposition is also expected to influence the NMC811 interface stability.^10,25^ To further investigate this, 60 h potential holds in 3 *m* electrolyte were performed to preform a CEI, and then, the electrodes were subjected to a second 60 h potential hold in 0.5 *m* electrolyte. In Figure 1b, a significantly lower average oxidation current density is seen for the sample with the preformed CEI compared to those without this preforming step. However, the oxidation current is still somewhat higher than that in the 3 *m* electrolyte case. Although some labile CEI components may be removed on electrode retrieval and cell reassembly, this nevertheless suggests that the oxidation current is strongly suppressed by the preformed CEI. The lower current densities observed at high salt concentrations are thus attributable to both the CEI that forms and the improved electrolyte stability.

### NMC811 Interfacial Characterization

**Figure 2:**
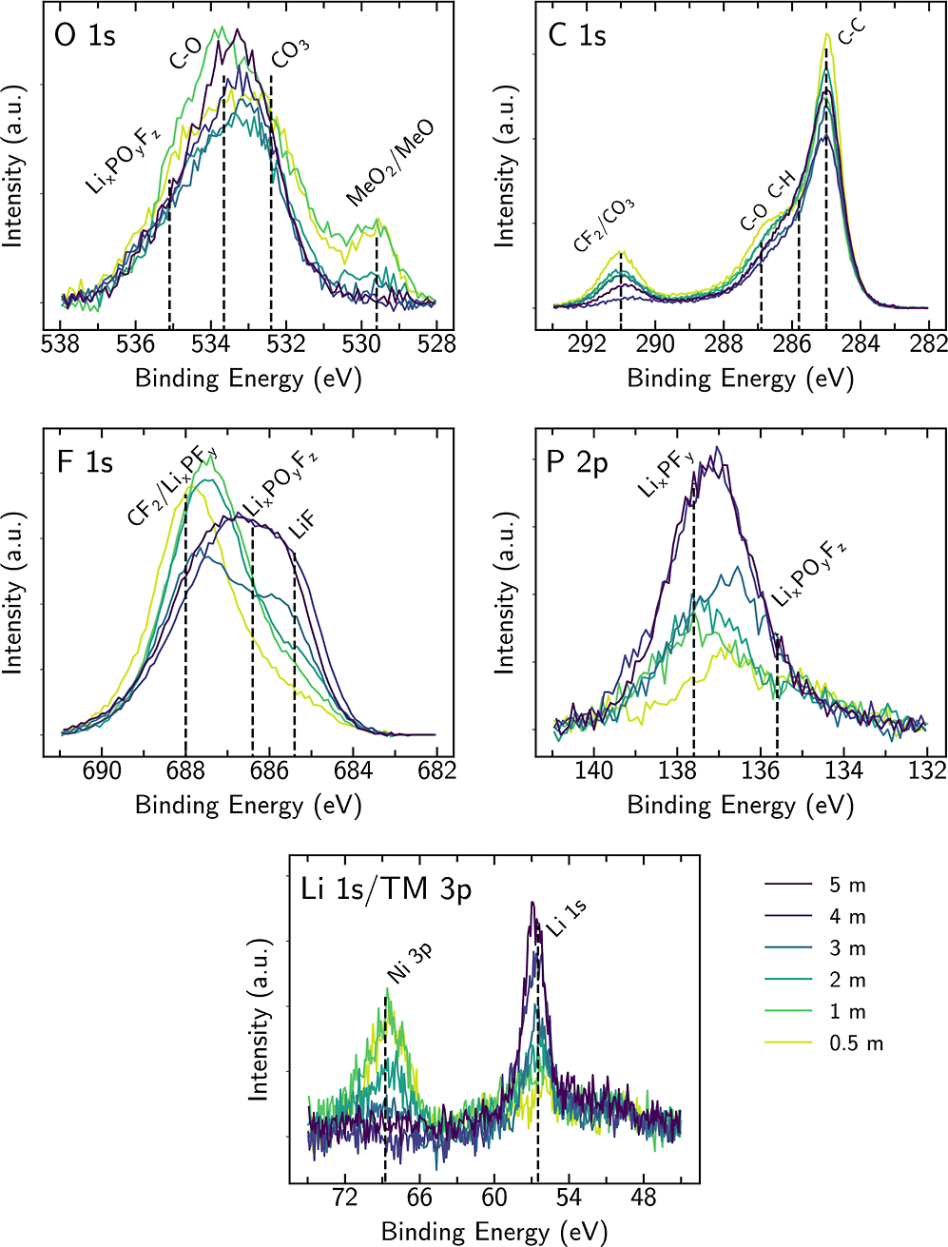
O 1s, C 1s, F 1s, P 2p, and Li 1s/TM 3p core level spectra of NMC811 electrodes for the different salt concentrations (0.5–5 *m*), following 60 h potential holds at 4.5 V and discharge to 3.8 V vs Li/Li^+^.

The composition of the CEI formed on the NMC811 electrodes following potential holds at the different salt concentrations was investigated using XPS, as shown in Figure 2. The same main spectral features are seen as those reported in our prior XPS study of cycled NMC811.^10^

The O 1s spectra show a clear decrease in the lattice oxygen peak (≈529.6 eV) with increasing salt concentration, indicating the formation of a thicker CEI.^10,41,42^ We note that this peak can have contributions from both the layered NMC811 (MeO_2_, Me = Ni, Co, Mn) and RSL (MeO-like), which both show similar peak positions. No clear trend with salt concentration is apparent for the other O 1s species. In the C 1s spectra, the intensities of peaks related to the PVDF binder (CF_2_, ≈291.0 eV; C–H, ≈285.8 eV)^10^ and carbon black (C–C, ≈285.0 eV)^41,43^ also decrease with increasing salt concentration, indicating that they are also attenuated by increasing CEI thickness. Similar to the O 1s spectra, the contributions of oxygen-containing species to the C 1s spectra do not show clear trends, although the slight decrease in C–O contributions (≈286.9 eV) at higher salt concentrations is consistent with a thicker CEI attenuating C–O surface species associated with carbon black or PVDF.

The F 1s spectra at lower electrolyte concentrations show strong PVDF binder contributions (CF_2_ ≈ 688.0 eV).^10^ Lower binding energy peaks, attributable to LiF (≈685.4 eV)^10,44^ and Li_*x*_PO_*y*_F_*z*_ (≈686.2 eV),^10^ grow in intensity with increasing salt concentration, indicating that more LiPF_6_ decomposition occurs for the higher concentration electrolytes. This is also supported by the P 2p spectra where the intensities of both the Li_*x*_PF_*y*_ feature (≈137.6 eV)^10,45,46^ and Li_*x*_PO_*y*_F_*z*_ feature (≈135.6 eV)^10,45,47^ increase with salt concentration.

In the Li 1s/TM 3p core level region, the Ni 3p peak (≈68.8 eV) intensity is seen to decrease with increasing salt concentration, while the Li 1s peak increases. This is consistent with a thicker CEI, rich in LiF/Li_*x*_PO_*y*_F_*z*_ forming with increasing salt concentration, covering the NMC811. Although the Mn and Co 3p core levels are also expected in this region (≈50.0 and ≈61.0 eV, respectively), they are not discernible above the noise due to their lower concentration in the cathode.

**Figure 3:**
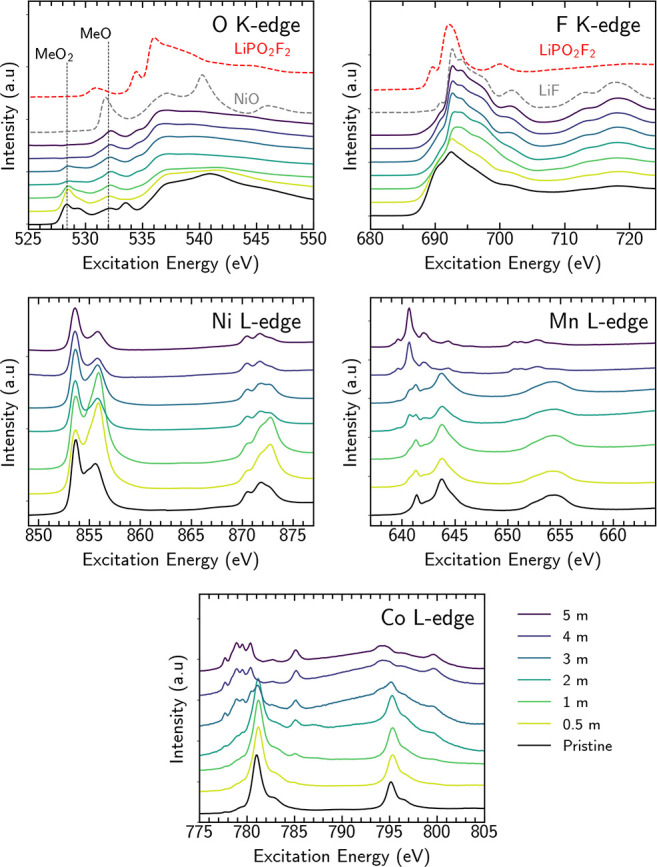
O K-edge, F K-edge, Ni L-edge, Mn L-edge, and Co L-edge TEY XAS spectra of NMC811 electrodes for the different salt concentrations (0.5–5 *m*), following 60 h potential holds at 4.5 V and discharge to 3.8 V vs Li/Li^+^. The pristine NMC811 electrode spectra are also included for reference.

The nature of the CEI and RSL formed at the NMC811 surface at the different salt concentrations was further investigated using XAS. Interface-sensitive (≈10 nm) TEY-XAS measurements of NMC811 electrodes in their pristine state and following 60 h potential holds in the different salt concentrations are shown in Figure 3. The pristine electrodes exhibit pre-edge features at 528.6 and 529.6 eV associated with O 1s core electron excitations into hybridized TM 3d–O 2p orbitals of the layered NMC811 (MeO_2_).^10,30,48^ The peak at 532.3 eV is attributable to transitions to hybridized TM 3d–O 2p orbitals of the RSL (MeO-like),^10,49^ and the peak at 533.8 eV is attributable to transitions to the π* orbital of the C=O group of Li_2_CO_3_. Li_2_CO_3_ is a common surface contaminant formed as a result of residual CO_2_ in the glovebox atmosphere, a reaction typically accompanied by some RSL formation.^10,30,48^ The features above 536 eV are primarily associated with transitions to hybridized TM 4s–O 2p and higher unoccupied orbitals of NMC811, although there are also contributions from Li_2_CO_3_ in this region.^50,51^ Simultaneously acquired bulk-sensitive FY-XAS (see Figure S3) shows only a weak RSL feature and no discernible Li_2_CO_3_ features, confirming that these are surface species.

TEY-XAS following the potential holds shows no discernible feature at 533.8 eV for all electrolyte concentrations, indicating that Li_2_CO_3_ is either electrochemically decomposed or chemically reacts with electrolyte decomposition products, or both.^16,52^ Most notably, the intensity of the MeO_2_ peaks is seen to decrease with increasing salt concentration and is barely apparent at electrolyte concentrations of ≥4 *m*. Decreased intensity of these features has previously been attributed to oxygen loss from the NMC811;^44^ however, as noted in our earlier work, thickening of the CEI layer must also be considered.^10^ Based on the information depth of the TEY-XAS, we estimate that a CEI thickness of ≥10 nm is required for the disappearance of the MeO_2_ peaks in the O K-edge and O 1s spectra.

The intensity of the MeO-like feature at 532.3 eV does not similarly decrease with increasing salt concentration and even slightly grows. Although this could suggest RSL thickening, which is expected for the NMC811 when cycled above 4.3 V vs Li/Li^+^ in standard 1 M electrolytes,^10,49^ there is no corresponding increase in the MeO-like feature seen with FY-XAS (see Figure S3). XPS also shows attenuation of the O 1s peak associated with both MeO_2_ and MeO with increasing salt concentration. Furthermore, additional contributions to the O K-edge spectra, particularly at 537.7 and 540.8 eV, would be expected for RSL growth (see the NiO reference spectrum). Instead, the slight increase in intensity at 532.3 eV coincides with the emergence of features at 534.7 and 536.2 eV for electrolyte concentrations ≥2 *m*. These correspond closely to features seen in the LiPO_2_F_2_ reference spectrum and for other oxyfluorides.^53,54^ Note that the peak at ≈531.0 eV in the LiPO_2_F_2_ TEY-XAS spectrum is not seen in the corresponding TFY-XAS spectrum, indicating that it relates to surface contamination. Phosphate-containing species, which XPS has indicated increasingly form at high salt concentrations, likely form alongside oxyfluorides and contribute to the peak at 532.3 eV.^55,56^ These observations suggest that CEI rather than RSL thickening is primarily responsible for the decreasing MeO_2_ peak intensity, with the increased formation of Li_*x*_PO_*y*_F_*z*_ species at high salt concentrations responsible for the growth in the features at 532.3, 534.7, and 536.2 eV.

The F K-edge spectrum of the pristine electrode can be assigned predominantly to PVDF, and this continues to contribute to the spectra measured for the lower concentration electrolytes. For the 1 and 2 *m* electrolytes, additional broad features are apparent, particularly in the range 693–705 eV, that are qualitatively similar to reported line shapes for oxyfluorides^54,57,58^ and the LiPO_2_F_2_ reference spectrum. For higher salt concentrations, sharp features are increasingly apparent at 692.7, 694.1, and 701.6 eV suggesting more LiF formation.^31^ This further confirms the XPS interpretation that a thicker CEI which is rich in LiF/Li_*x*_PO_*y*_F_*z*_ forms at higher salt concentrations.

The Ni L-edge spectra exhibit sharp features in the L_3_ edge at 853.7 and 856.0 eV. The lower energy feature is primarily attributable to Ni^2+^, and growth in the intensity of the higher energy feature reflects higher Ni-oxidation states with this feature dominating for Ni^4+^.^7,10,48,59,60^ In the Mn L-edge, the peaks at 641.4 and 643.8 eV are assigned to Mn^4+^. The features observed at 639.6 and 642.1 eV have contributions from Mn^3+^ and Mn^2+^, while the large feature at 640.7 eV indicates Mn^2+^.^10,61,62^ In the Co L-edge, the peak at 781.0 eV is attributed to Co^3+^ in the low spin state with the peaks at 777.7, 778.9, 779.5, and 780.4 eV attributable to Co^2+^ in the high spin state.^7,10,48,59^ The Ni, Mn, and Co L-edges thus suggest that the TM species follow similar trends with increasing salt concentration. At lower salt concentrations, the probed TM centers near the NMC811 surface are predominantly in their more oxidized form (+3 formal oxidation states for Ni and Co, +4 for Mn), while for higher salt concentrations Me^2+^ species increasingly contribute, with these dominating at the highest concentrations.

From Figure 2, it is apparent that the peaks associated with the NMC811 active material are strongly attenuated by CEI formation, and given the similar information depth of TEY-XAS (≈10 nm), the changes in the TM L-edges at higher electrolyte concentrations are attributable to more reduced TM species close to the surface of the NMC811, or incorporated into the CEI itself. More bulk-sensitive (≈100 nm) FY-XAS (Figure S3) measurements show no corresponding increase in the MeO feature in the O K-edge or more reduced species in the TM L-edges. Indeed, the Ni L-edge measured in FY shows a noticeable drop in the intensity of the 853.7 eV feature for electrolyte concentrations ≥4 *m*. This suggests that rather than a more densified/thick RSL at higher electrolyte concentrations, as might be assumed based on the TEY-XAS alone, the TEY-XAS features arise from relatively low concentrations of TM^2+^ ions at the very surface of the NMC811 and dispersed within the thicker CEI, at concentrations where they are not readily detected in the XPS measurements.

Interestingly, the Co L-edge TEY-XAS of the higher concentration electrolytes shows additional features at 785.2 and 799.7 eV (Figure 3), which are not seen in the FY-XAS (Figure S3) or reference measurements of bulk CoO. These features have previously been attributed to Co coordinating certain ligand molecules and the associated metal-to-ligand charge transfer (MLCT).^63,64^ The growth in intensity of these features with electrolyte concentration seen herein would potentially be consistent with the trapping of dispersed Co^2+^ in the CEI and coordination with P_*x*_O_*y*_F_*z*_ species. However, reports of similar features without the presence of Co have cast doubt on the attribution of these features,^65^ and they may instead relate to the Ba M_4,5_-edge which exhibits a very similar spin–orbit splitting.^66,67^ Indeed, XPS measurements of the Ba 3d core level for the GF separator (Figure S7) confirm the presence of Ba. Moreover, the growth in intensity of the 785.2 and 799.7 eV features with electrolyte concentration correlates closely with other evidence of separator degradation (see below).

### Soluble Electrolyte Decomposition Products

**Figure 4:**
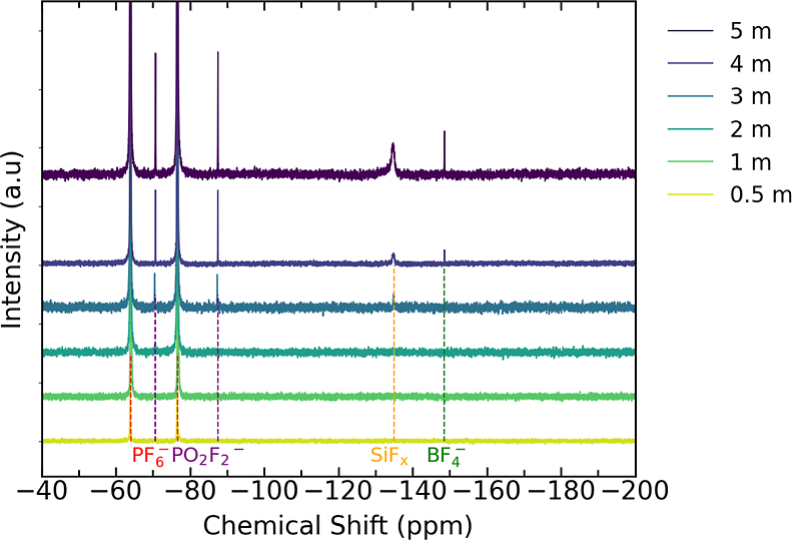
^19^F NMR spectra of the different electrolyte concentrations following 60 h potential holds at 3.8 V vs Li/Li^+^ for the different salt concentrations (0.5–5 *m*).

Soluble degradation products in the electrolyte extracted from NMC811/LTO cells after 60 h of potentiostatic hold were investigated by ^19^F NMR spectroscopy. From Figure 4, a doublet centered at −70.5 ppm (d, ^1^*J*_P–F_ = 710 Hz) is seen for all salt concentrations which corresponds to the presence of the PF_6_^–^ ions. At salt concentrations of ≥3 *m*, a doublet centered at −78.5 ppm (d, ^1^*J*_P–F_ = 955 Hz) alongside singlets at −134.9 ppm (s) and −148.6 ppm (s) emerge attributable to PO_2_F_2_^–^, SiF_*x*_, and BF_4_^–^ respectively.^16,68^ SiF_*x*_ and BF_4_^–^ are expected to form by the reaction of HF in the electrolyte with the GF separator.^16,68^ The stronger intensity of these features reflects that more separator degradation occurs in the higher concentration electrolytes, indicating that more HF is generated. We note that HF is not discernible in the ^19^F (s, −171.7 ppm)^16,68,69^ or ^1^H (Figure S5) NMR spectra for any of the electrolyte concentrations. This suggests that the HF formed is continuously consumed by reaction with species present in the cells rather than accumulating to high levels.

### Transition Metal Dissolution and Crossover

**Figure 5:**
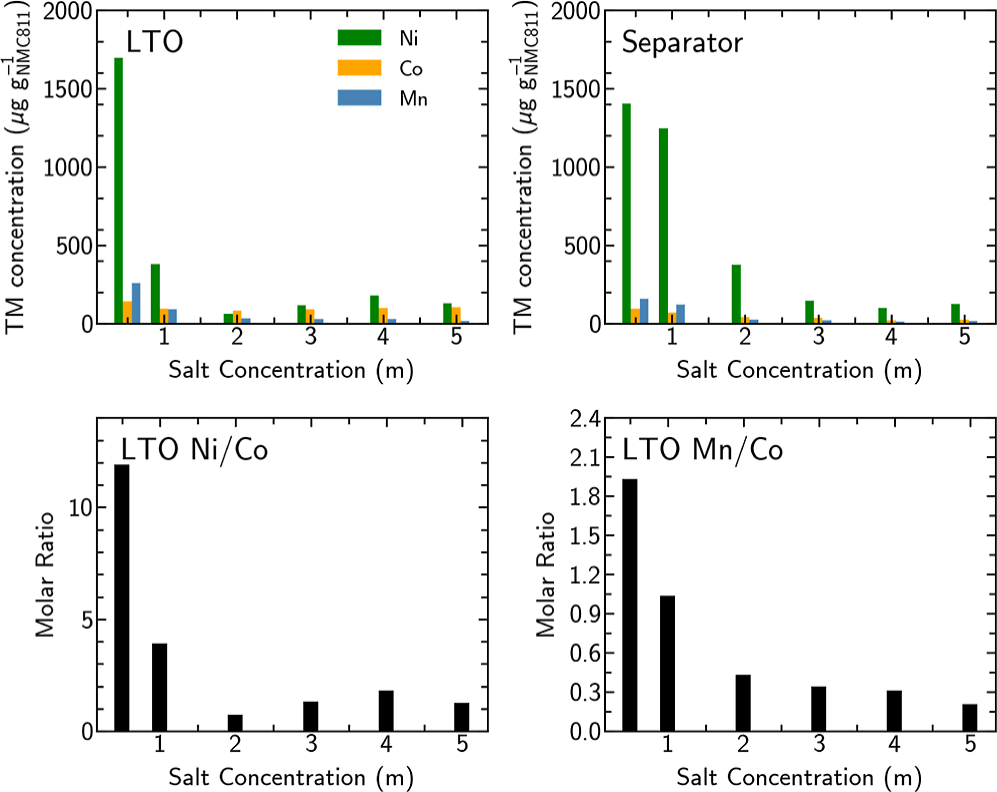
ICP-OES of LTO counter electrode and GF separator following 60 h potential holds and molar ratios for deposited TMs on the LTO electrode for the different salt concentrations (0.5–5 *m*).

To investigate the extent of TM dissolution from the cathode as a result of the potential holds, the LTO anode and GF separator were extracted from the coin cells for ICP-OES measurements. Figure 5 shows the concentrations of Co, Mn, and Ni deposited on the LTO electrode and accumulated in the separator. The concentrations of dissolved TMs in both the LTO electrode and the separator are seen to decrease significantly for the higher concentration electrolytes. The drop-off in TM concentration with electrolyte salt concentration is more rapid for the LTO electrode than for the GF separator, suggesting that the higher salt concentration not only suppresses TM dissolution from the cathode (sum of LTO and separator TM concentrations) but also influences TM deposition at the LTO electrode.

Considering the molar ratios of Ni/Co and Mn/Co in the LTO electrodes, the amount of Ni and Mn crossover is found to be more strongly suppressed compared to Co at higher salt concentrations, with ratios well below the 8:1 and 1:1 expected based on the cathode composition. The molar ratios of TMs in the GF separator show a similar trend with lower Ni/Co and Mn/Co ratios for the higher concentration electrolytes (see Figure S3); however, the differences are less pronounced than for the LTO. Thus, for the higher concentration electrolytes, not only do Ni and Mn appear to dissolve less readily from the cathode than Co, but their incorporation from the electrolyte into the LTO electrode is also more suppressed.

## Discussion

The 60 h potential holds performed here reveal the dependence of electrolyte stability on salt concentration at high potentials (Figure 1), with the oxidation current associated with electrolyte decomposition suppressed at higher salt concentrations. From Figure 1b, it is apparent that preforming the CEI in a higher concentration (3 *m*) electrolyte partially suppresses the oxidation current observed in a 0.5 *m* electrolyte, confirming the important role of the CEI. However, the current is still somewhat higher than that seen during the 3 *m* sample hold, suggesting that stabilization of the solvent components in higher concentration electrolytes also plays a role. Furthermore, lower *R*_ct_ values are observed with the higher concentration electrolytes at high voltages, indicating less extensive RSL formation.

**Figure 6:**
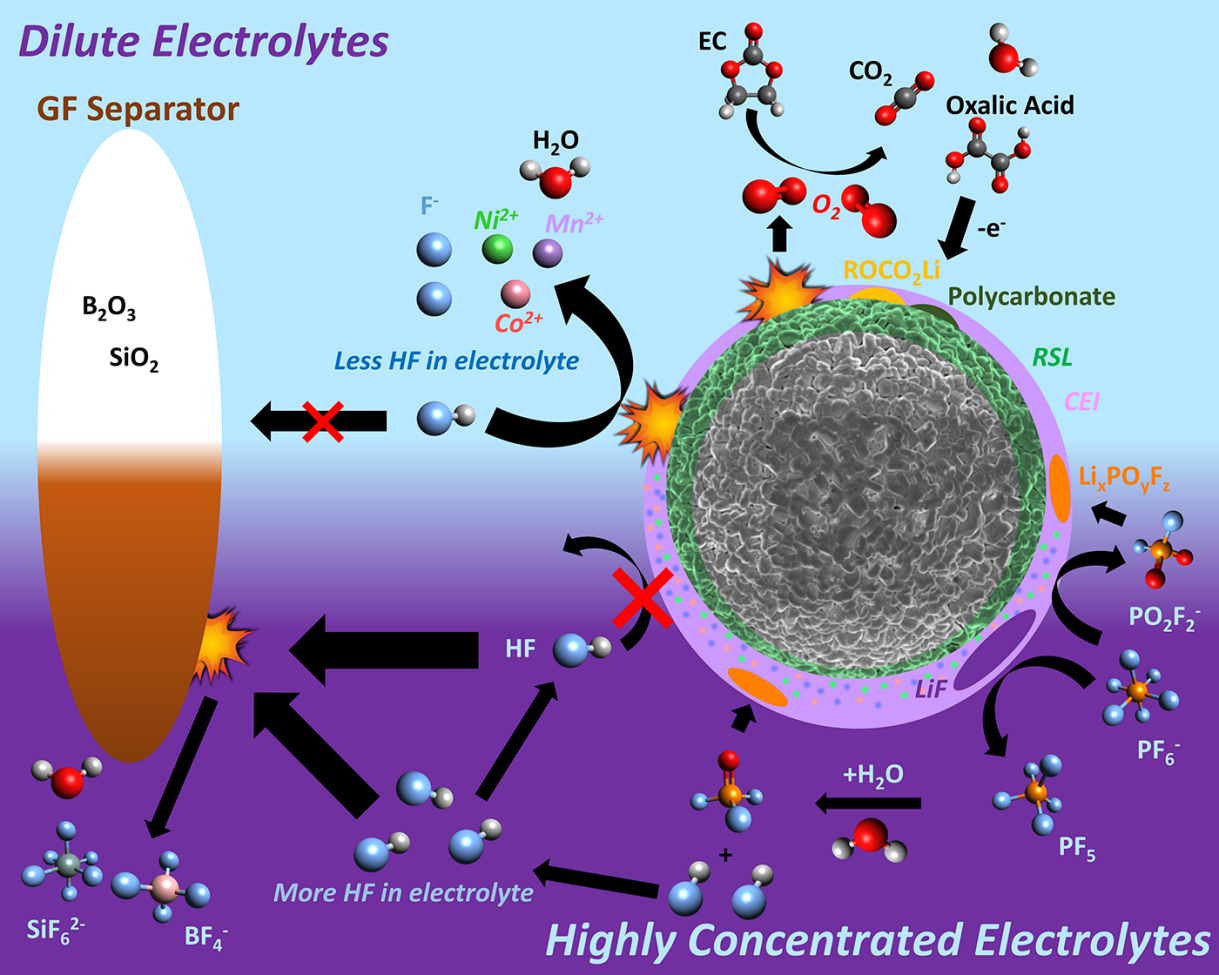
Illustration of the reaction mechanisms between NMC811 and the electrolyte.

In order to rationalize these improvements in electrolyte stability, and *R*_ct_ values, we now consider the results of detailed chemical characterization of the cathode, electrolyte, and species that cross over to the anode in the context of prior literature. Figure 6 summarizes how the contributions from key chemical processes differ between the lower and higher concentration electrolytes.

For NMC811 in lower concentration electrolytes, significant lattice oxygen release and accompanying RSL thickening are expected for the hold potentials used herein (4.5 V vs Li/Li^+^).^1,5,8−10,13,14^ Consistent with this, TEY-XAS shows a MeO feature at 532.3 eV in the O K-edge, and potential-dependent EIS measurements show a rapid rise in *R*_ct_ at quite low potentials reflecting the limited availability of Li vacancies due to significant RSL formation.^7,11,12^ Release of ^1^O_2_ from NMC at high potentials^1,8−10,13,14^ has been implicated in chemically oxidizing EC and linear carbonate molecules to yield polycarbonate and ROCO_2_Li species atop the RSL.^16,68^ The XPS measurements herein are consistent with the presence of such species for all electrolyte concentrations studied (Figure 2). At low LiPF_6_ concentrations, XPS and XAS reveal that the CEI is relatively thin with limited LiF content, and NMR indicates that minimal electrolyte salt degradation occurs. This thin, organic-rich CEI allows HF and other acidic species, such as H_3_PO_4_, to attack the RSL leading to TM dissolution into the electrolyte and crossover to the anode as seen with ICP-OES. MeO can react with various acidic species forming water and Me^2+^ species.^70,71^
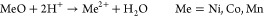
2

For higher LiPF_6_ concentrations, XPS and XAS reveal that a much thicker CEI is formed that is rich in Li_*x*_PO_*y*_F_*z*_, and increasingly LiF at the highest concentrations. Strikingly similar CEI compositions are observed for the 4 and 5 *m* electrolytes, distinct from those seen at lower salt concentrations. Furthermore, comparable degrees of oxidation current passivation are seen for the ≥3 *m* electrolytes, suggesting a transition in behavior around this concentration. This coincides with the concentration at which the proportion of free-solvent molecules becomes very low, with the formation of contact–ion pairs and ion aggregates dominating.^72,73^ Li^+^ coordination has been reported to lower the highest occupied molecular orbital (HOMO) energies of solvent molecules, increasing their oxidative stability.^74−77^ We therefore suggest that the change in the solvation structures present at ≥3 *m* favors PF_6_^–^ anion decomposition over solvent decomposition, leading to a more Li_*x*_PO_*y*_F_*z*_/LiF-rich CEI. The greater CEI thickness seen at higher concentrations is consistent with the increased chemical potential of the PF_6_^–^ resulting in more LiF formation atop the RSL alongside the formation of PF_5_.^16,78−80^
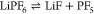
3

The attenuation of TM core levels in XPS for the higher concentration electrolytes indicates that the overall amount of TMs in the thicker CEIs is low, with TEY-XAS showing an increased proportion of Me^2+^ species. More bulk-sensitive FY-XAS also indicates a lesser extent of RSL formation, consistent with the formation of MeF_2_ and Me_*x*_PO_*y*_F_*z*_ at the outer RSL or within the thick CEI. The formation of these components can be attributed to the increased reaction of the outer RSL with LiPF_6_ and PF_5_, and other products of these reactions are indeed seen with XPS (LiF) and NMR (PO_2_F_2_^–^).
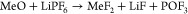
4
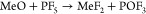
5
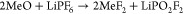
6

Interestingly, LiPO_2_F_2_ is increasingly considered as an electrolyte additive for stabilizing high-voltage cathode materials due to its ability to form a passivating CEI,^81,82^ yielding insoluble Li_*x*_PO_*y*_F_*z*_ species^83−86^ as well as Me_*x*_PO_*y*_F_*z*_, as evidenced here in the TEY-XAS of the higher concentration electrolytes.

PO_*y*_F_*z*_-species can also be produced by increased LiPF_6_ hydrolysis at the higher salt concentrations, related to trace moisture in the electrolyte, including that arising from solvent molecule oxidation.^16,80^
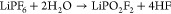
7
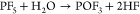
8

These PO_*y*_F_*z*_-species can be further hydrolyzed to produce other acidic species, such as H_3_PO_4_,^87^ in addition to reacting with the cathode surface to yield insoluble Li_*x*_PO_*y*_F_*z*_ components of the CEI. These acidic species, including HF, have already been discussed above in the context of TM dissolution.

Important to the improved stability seen for the higher concentration electrolytes is the protection afforded by the thicker Li_*x*_PO_*y*_F_*z*_- and LiF-rich CEI formed, which leads to thinner/less-densified RSLs with lower *R*_ct_ values, less TM dissolution, and lower oxidation currents at high potentials. EC from the electrolyte has been suggested to enhance oxygen loss from NMC leading to more cathode surface degradation.^5^ For higher concentration electrolytes, the lesser extent of RSL formation seen by FY-XAS may relate to the reduced access of EC to the NMC surface as well as the increased stabilization of EC in higher concentration electrolytes. The thicker CEI is also seen to limit RSL attack by acidic species, with much lower TM dissolution and crossover observed by ICP-OES. This inevitably means HF consumption at the cathode is suppressed, and thus, more HF is available to degrade the GF separator in the higher concentration electrolytes, accounting for the higher concentrations of BF_4_^–^ and SiF_*x*_ species observed in NMR.^16,68^
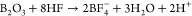
9
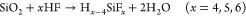
10

It should however be noted that GF separators are not widely used in commercial LIBs and thus, in this context, this form of degradation may be less relevant.

## Conclusions

In this work, new understanding of the effect of LiPF_6_ salt concentration on the interfacial reactivity of charged Ni-rich NMC cathodes in carbonate-based electrolytes is unveiled by combining electrochemical measurements with chemical analysis of the cathode interfaces and electrolytes. The degree of electrolyte oxidation at Ni-rich NMC electrodes is found to be suppressed by increasing salt concentration. The higher concentration electrolytes lead to formation of more Li_*x*_PO_*y*_F_*z*_-/LiF-rich CEIs which limit the access of solvent molecules and acidic species to the cathode surfaces resulting in thinner RSLs with lower charge transfer impedances, less TM dissolution into the electrolyte, and lower parasitic oxidation currents observed at high potentials. The understanding developed of the composition and structure of the beneficial CEI formed is expected to inform the rational design of new electrolyte formulations which can stabilize the cathode electrolyte interface, which is a crucial step toward enabling the use of high-voltage Ni-rich cathodes in commercial LIBs. This includes the identification of suitable, low-cost additives to achieve similar improvements in stability to those observed with highly concentrated electrolytes, without sacrificing the benefits of lower concentration LiPF_6_-based electrolytes, that is, lower salt cost, higher ionic conductivity, and fewer negative environmental impacts.

## Supporting Information Available

Cathode–electrolyte interfacial impedance example plots and theory, FY-XAS of NMC811 electrodes following potential holds, GF separator molar ratios determined from ICP-OES, ^1^H NMR spectra of the electrolyte following potential holds, current densities during potential holds for two-electrode coin cells, and XPS measurements of the Ba 3d core level for the pristine GF separator (PDF)
