# Supplementary material for: Role of Salt Concentration in Stabilizing Charged Ni-Rich Cathode Interfaces in Li-Ion Batteries
Source: Chem Mater. 2024 Mar 26;36(7):3334–44. doi: 10.1021/acs.chemmater.4c00004 (PMC11008099; doi:10.1021/acs.chemmater.4c00004)
Supplement: Supplementary file 1 — cm4c00004_si_001.pdf [file cm4c00004_si_001.pdf]

Supplementary Information: Role of Salt Concentration in Stabilizing Charged Ni-Rich Cathode Interfaces in Li-ion Batteries

Conor M. E. Phelan,[†] Erik Björklund,[†,‡] Jasper Singh,[†] Michael Fraser,[†,‡] Pravin N. Didwal,[†,‡] Gregory J. Rees,[†,‡] Zachary Ruff,[¶,‡] Pilar Ferrer,[§] David C. Grinter,[§] Clare P. Grey,[¶] and Robert S. Weatherup[*,†,§,‖,‡]

†*Department of Materials, University of Oxford, Parks Road, Oxford OX1 3PH, United Kingdom*

‡*The Faraday Institution, Quad One, Harwell Science and Innovation Campus, Didcot OX11 0RA, UK*

¶*Department of Chemistry, University of Cambridge, Lensfield Road, CB2 1EW, Cambridge, UK*

§*Diamond Light Source, Didcot, Oxfordshire OX11 0DE, United Kingdom*

‖*Research Complex at Harwell, Didcot, Oxfordshire OX11 0DE, United Kingdom*

E-mail: robert.weatherup@materials.ox.ac.uk

Contents

Cathode-electrolyte interfacial impedance **3**

NMC811 - FY-XAS **6**

ICP-OES - GF Separator Molar Ratios **8**

^{1}H NMR **9**

Coin Cell 60 h Hold **10**

GF Separator XPS **11**

Cathode-electrolyte interfacial impedance

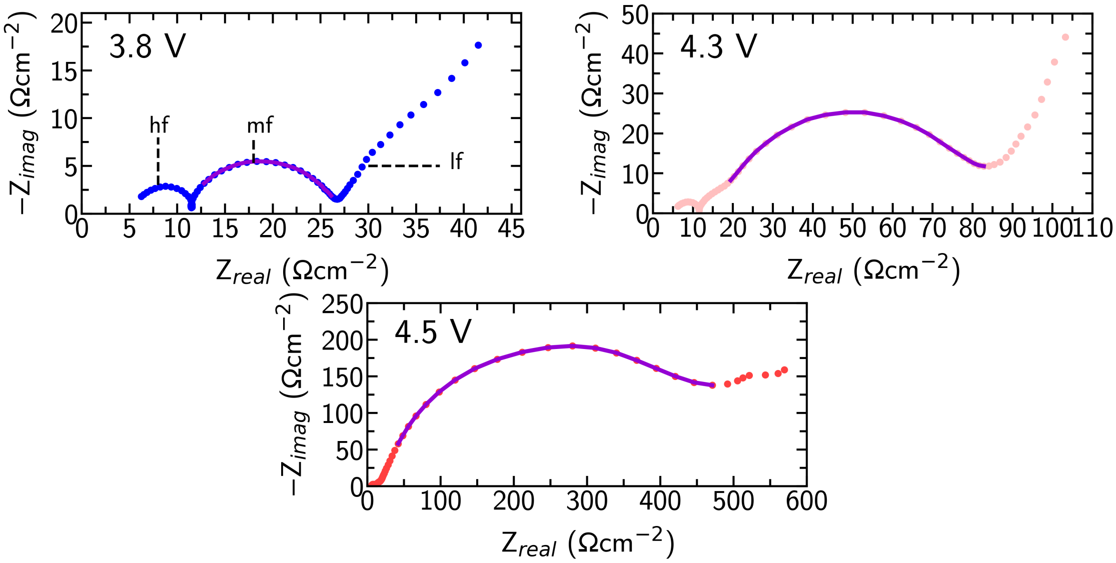

Figure S1: Example Nyquist plots of the NMC811 cathode at various potentials vs. Li/Li^+ measured in the three-electrode swagelok cell of NMC811/LTO with Li metal as reference for the 1 m LP57 electrolyte. To extract the charge transfer resistance, R_{ct} from the measured Nyquist plots, the mid-frequency (mf) semicircle was fitted using the equivalent electrochemical circuit from Figure 1 (c) from the main text. The purple line shows the fit of the equivalent circuit from Figure 1 (c) to the experimental data from the main text.

To assist in interpreting the variations in R_{ct} observed, the intercalation kinetics of battery electrodes can first be considered using the Butler-Volmer equation:

$$i = i_0 \left[exp(\frac{\alpha_a F(\phi_1 - \phi_2 - U)}{RT}) - exp(-\frac{\alpha_c F(\phi_1 - \phi_2 - U)}{RT}) \right] \tag{1}$$

where i_0 is the exchange current density, ϕ_1 is the electrical potential in the cathode, ϕ_2 is the electrical potential in the electrolyte, U is the open circuit voltage of the cathode interface, F is Faraday's constant, R is the universal gas constant, with α_a and α_c the anodic and cathodic transfer coefficients respectively. Newman and coworkers assumed the intercalation kinetics of battery electrodes to be a first-order process and thereby obtained an expression for i_0 in a liquid electrolyte:[1–3]

$$i_0 = Fk(c_{s,max} - c_s)^{\alpha_a} c_s^{\alpha_c} c_e^{\alpha_a} \tag{2}$$

where c_e is the local electrolyte Li^+ concentration, c_s is the concentration of Li in the solid electrode, $(c_{s,max} - c_s)$ is the concentration of unoccupied sites in the electrode, and the rate constant k can be described by the Arrhenius equation with an appropriate pre-exponential factor and activation energy.

For concentrated electrolytes, the limited Li solubility in the electrolyte must also be considered, as has been recently highlighted.[4] A similar approach to that employed by Newman and coworkers for polymer electrolytes can be taken,[2] by adapting the expression for i_0 to:

$$i_0 = Fk(c_{s,max} - c_s)^{\alpha_a} c_s^{\alpha_c} (c_{e,max} - c_e)^{\alpha_c} c_e^{\alpha_a} \tag{3}$$

where the solubility limit of the electrolyte is taken into account by $(c_{e,max} - c_e)$. Assuming small perturbations ($\leq$5 mV) of the overpotential, $\eta = \phi_1 - \phi_2 - U$, the expression for i may be linearized using exp(x) $\approx$ 1 + x and expressed as:

$$i = \frac{Fi_0}{RT}\eta \tag{4}$$

The charge transfer resistance, R_{ct}, may thus be expressed as:

$$R_{ct} = \lim_{\eta \to 0} \frac{\eta}{i} = \frac{RT}{Fi_0} = \frac{RT}{F^2 k(c_{s,max} - c_s)^{\alpha_a} c_s^{\alpha_c} (c_{e,max} - c_e)^{\alpha_c} c_e^{\alpha_a}} \tag{5}$$

Figure S2 shows the variation in R_{ct} with electrolyte molality (a) and molarity (b) for pristine NMC811 electrodes directly cycled to 3.8 V, 3.9 V, and then 4.0 V, based on room temperature EIS measurements at each of these potentials such that there is very limited RSL and CEI formation. For these conditions, $c_{s,max}$, c_s and $c_{e,max}$ can be considered constant and given that the 5 m electrolyte is found to be very close to saturation, we estimate $c_{e,max} \approx$ 4.4 M. Equation 5 is thus sufficient to qualitatively capture the drop in R_{ct} with electrolyte concentration seen below 3 m ($\approx$ 3 M) and the increase in R_{ct} seen above this. Although not necessary for our analysis herein, the asymmetry apparent in figure S6 (b) indicates that the common assumption that $\alpha_a = \alpha_c$ is unlikely to be valid, and indeed more

quantitative analysis may require the kinetic transfer coefficients for the liquid and solid phases to be treated separately, as suggested in the analysis of concentrated electrolytes by Morasch et al.[4]

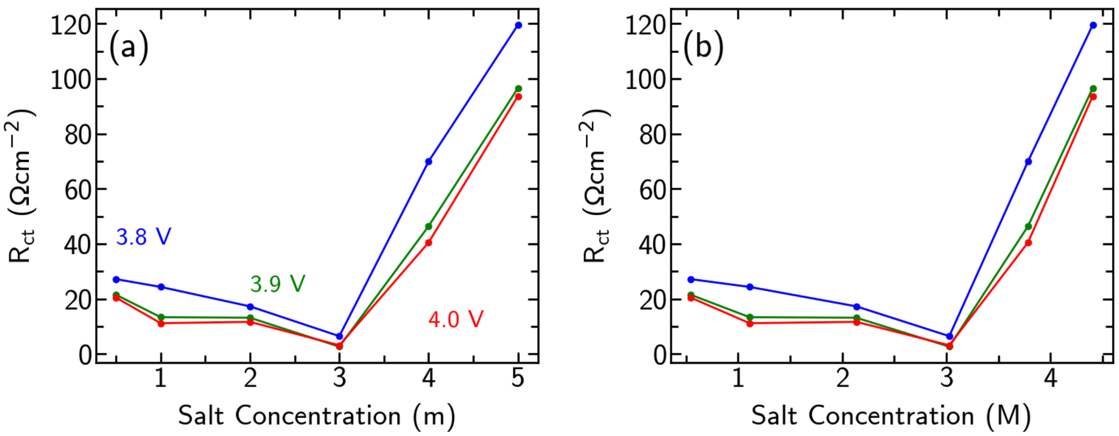

Figure S2: Variation in R_{ct} with (a) electrolyte molality and (b) electrolyte molarity for pristine NMC811 electrodes directly cycled to 3.8 V, 3.9 V, and then 4.0 V.

NMC811 - FY-XAS

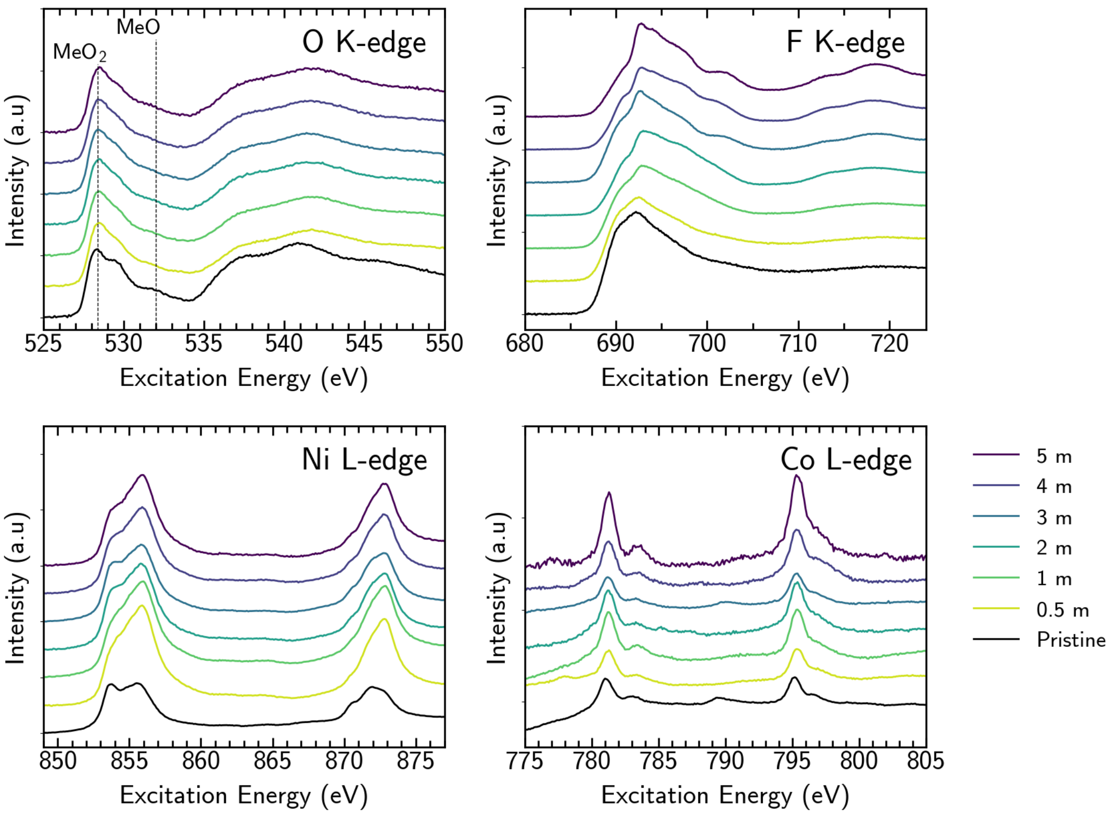

Figure S3: O K-edge, F K-edge, Ni L-edge and Co L-edge FY-XAS spectra of NMC811 electrodes following 60 h potential holds at 3.8 V vs. Li/Li^{+} for the different salt concentrations (0.5 m - 5 m).

The FY-XAS spectra of NMC811 electrodes after potential holds in the different salt concentrations are shown in Figure S3. The O K-edge spectra of the held electrodes exhibit a MeO_2 feature of similar intensity at all concentrations with no significant MeO feature. This confirms that the suppression of the MeO_2 feature at higher electrolyte concentrations seen in TEY-XAS (Figure 3, main text) is a surface effect. Although the pristine electrode exhibits pre-edge features at 528.6 eV to the 529.6 eV, the 529.6 eV feature is less distinct in the potential held electrodes, consistent with their higher state of charge (discharged to 3.8 V vs. Li/Li^{+} or $Li_{1-x}TMO_2$ x = 0.44). The F K-edge spectrum becomes more LiF-like with increasing salt concentration similar to the TEY-XAS indicating a thick LiF layer forming on the NMC811 particle at the higher salt concentrations. The Ni and Co L-edges appear

to remain predominantly in their oxidised state (+3) with increasing salt concentration, indicating the change in oxidation state observed in the TEY is due to RSL formation rather than bulk oxidation state changes. For the 2 m and 3 m electrolytes, the Ni L-edge shows a more pronounced low-energy shoulder at 853.7 eV compared to the 4 m and 5 m, suggesting a lower extent of RSL formation at the surface of the NMC811 in the higher concentration electrolytes. The Mn L-edge suffers from severe distortions associated with saturation and self-absorption effects in FY mode and has thus not been included.

ICP-OES - GF Separator Molar Ratios

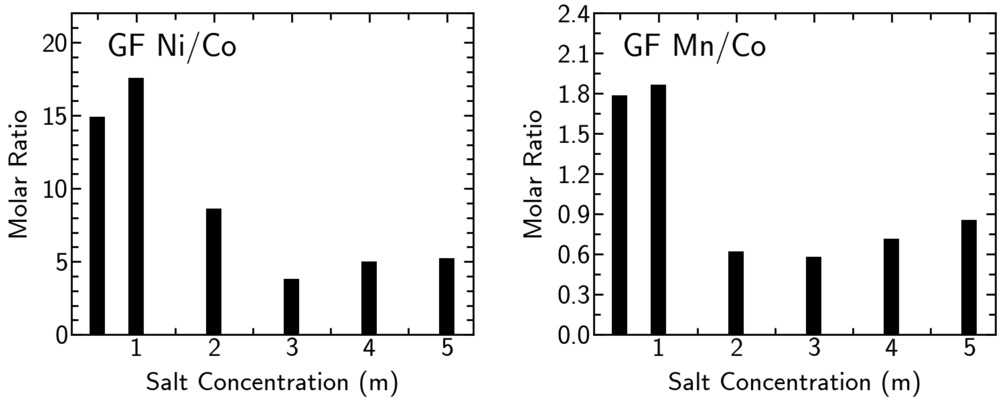

Figure S4: Molar ratios for accumulated TMs in the GF separator calculated based on Figure 5 in the main text.

At higher salt concentrations larger amounts of Ni are present relative to Co and Mn, indicating that Co^{2+} and Mn^{2+} are less soluble in the electrolyte than Ni^{2+}.

^{1}H NMR

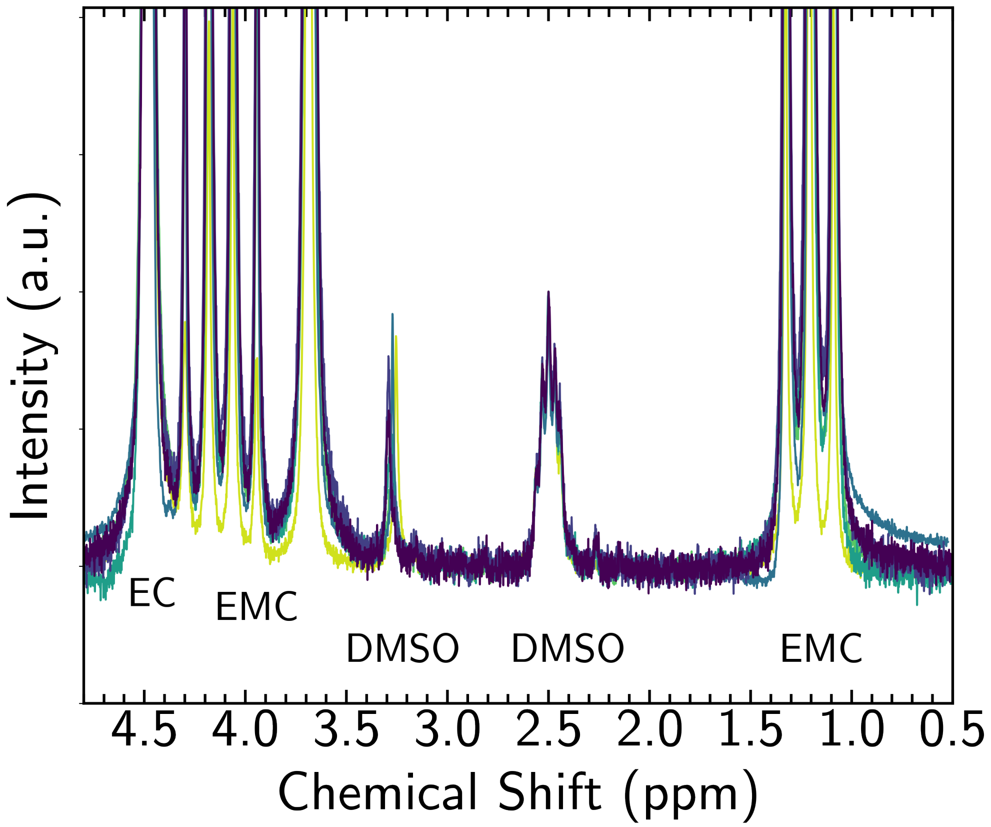

Figure S5: ^{1}H NMR spectra of the different electrolyte concentrations following 60 h potential holds at 3.8 V vs. Li/Li^{+} for the different salt concentrations (0.5 m - 5 m).

Coin Cell 60 h Hold

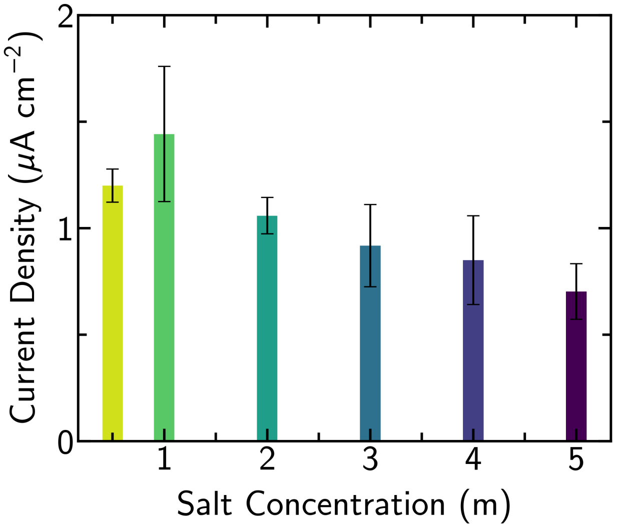

Figure S6: Average oxidation current density during final 20 h of potential holds from two or more cells including errorbars in coin cell format.

In contrast to the three-electrode Swagelok cell data shown in Figure 1 of the main text, the coin cell data show lower current densities for the lower concentration electrolytes, with the 0.5 m sample exhibiting a similar current density to the 1 m sample. We suggest this relates to the smaller electrolyte volumes used in the coin cells (60 μm, rather than 120 μm), with the lower concentration electrolytes where solvent decomposition dominates, being more readily saturated with solvent decomposition products resulting in a suppression of the parasitic oxidation currents.

GF Separator XPS

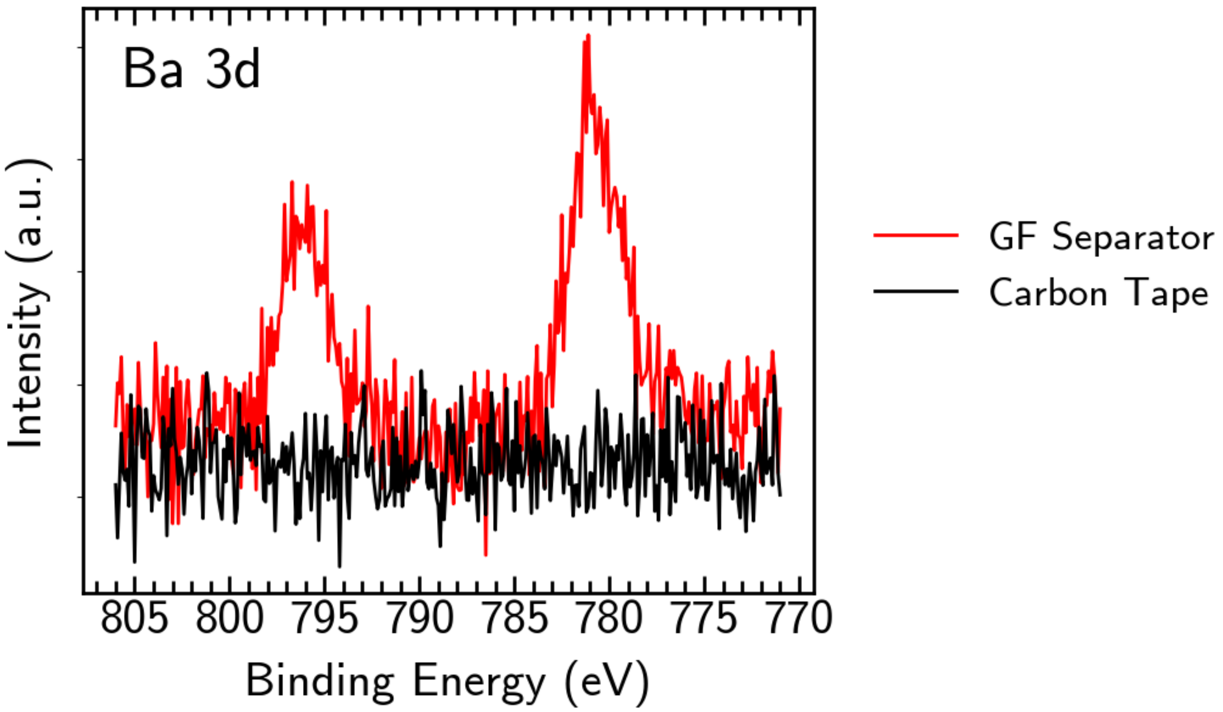

Figure S7: Ba 3d core level XP spectra of the pristine GF separator, and the carbon tape support used for mounting on the sample stage.

XPS measurements of the Ba 3d core level for the GF separator confirm the presence of Ba. Data for the carbon tape used to mount the separator on the sample stage was also recorded to confirm that the Ba 3d signal originates from the GF separator. To account for charging, the binding energies for the GF separator and the carbon tape were calibrated such that the adventitious carbon peak in the C 1s core levels was at 285.0 eV.

References

(1) Fuller, T. F.; Doyle, M.; Newman, J. Simulation and Optimization of the Dual Lithium Ion Insertion Cell. *Journal of The Electrochemical Society* **1994**, *141*.

(2) Doyle, M.; Fuller, T. F.; Newman, J. Modeling of Galvanostatic Charge and Discharge of the Lithium/Polymer/Insertion Cell. *Journal of The Electrochemical Society* **1993**, *140*.

(3) Newman, J.; Thomas-Alyea, K. E. *Electrochemical systems. Third edition*; John Wiley & Sons, Inc., 2004; p 205.

(4) Morasch, R.; Gasteiger, H. A.; Suthar, B. Li-Ion Battery Active Material Impedance Analysis I: Comparison of Measured NCM 111 Kinetics with Butler-Volmer Equation Based Predictions. *Journal of The Electrochemical Society* **2023**, *170*.
